# Fast-Activated Minimal Gated Unit: Lightweight Processing and Feature Recognition for Multiple Mechanical Impact Signals

**DOI:** 10.3390/s24165245

**Published:** 2024-08-14

**Authors:** Wenrui Wang, Dong Han, Xinyi Duan, Yaxin Yong, Zhengqing Wu, Xiang Ma, He Zhang, Keren Dai

**Affiliations:** 1 School of Mechanical Engineering, Nanjing University of Science and Technology, Nanjing 210094, China; wwr3099898032@njust.edu.cn (W.W.); duanxinyi@njust.edu.cn (X.D.); 10074617@njust.edu.cn (Y.Y.); 1219823401@njust.edu.cn (Z.W.); hezhangz@njust.edu.cn (H.Z.); 2 The Third Military Representative Office of the Army Armament Department in Nanjing, Nanjing 210000, China; 13851520355@139.com

**Keywords:** multiple dynamic impact signals, wavelet transform, lightweight network, signal identification

## Abstract

Multiple dynamic impact signals are widely used in a variety of engineering scenarios and are difficult to identify accurately and quickly due to the signal adhesion phenomenon caused by nonlinear interference. To address this problem, an intelligent algorithm combining wavelet transforms with lightweight neural networks is proposed. First, the features of multiple impact signals are analyzed by establishing a transfer model for multiple impacts in multibody dynamical systems, and interference is suppressed using wavelet transformation. Second, a lightweight neural network, i.e., fast-activated minimal gated unit (FMGU), is elaborated for multiple impact signals, which can reduce computational complexity and improve real-time performance. Third, the experimental results show that the proposed method maintains excellent feature recognition results compared to gate recurrent unit (GRU) and long short-term memory (LSTM) networks under all test datasets with varying impact speeds, while its metrics for computational complexity are 50% lower than those of the GRU and LSTM. Therefore, the proposed method is of great practical value for weak hardware application platforms that require the accurate identification of multiple dynamic impact signals in real time.

## 1. Introduction

With the advancement of modern industrial and military technologies, the issue of multiple mechanical impact loads with repetitive impulses has become widely discussed in industries such as aerospace [1], transportation [2], and the military [3]. Examples include the docking between aircraft and service vehicles, vehicle impact testing, and ammunition penetration into multi-layered targets [4,5,6]. With respect to vehicle impact testing and ammunition penetration, there are certain similarities and differences. The similarities are that both processes consist of multiple components, have complex dynamic transmission processes, and are affected by nonlinear noise during impacts, making it challenging to accurately identify the number of impacts. The differences are that, for ammunition penetration, the internal space is compact, leading to significant interference from the repeated transmission of shock waves. In contrast, vehicle systems have more internal voids and interference during impacts is severe due to structural deformation. There is great economic and strategic value in the feature identification and impact counting of these multiple-impact loads [7]. For example, identifying the number and magnitude of repetitive impacts during vehicle impact testing is critical for establishing a basic dataset for evaluating the safety performance of automobiles. Similarly, for penetration of multi-layer buildings, it is essential to accurately identify the number of consecutive impacts to achieve layer-specific ammunition explosions and effective damage. Unfortunately, these impact signals have strong nonlinear noise and interference, which makes it very challenging to perform signal feature identification in real time [8,9,10].

Traditionally, these multiple impact signals are first processed with wavelet transform, Fourier transform, etc., and then passed through a filter for identification [11,12]. In this framework, the effectiveness of feature recognition is strongly related to the design of the specific parameter sets of the filter, and they, in turn, depend on the frequency and amplitude of multiple impact signals and noise, respectively. Unfortunately, such prior information, with respect to multiple impact signals and noise, is unpredictable and variable in practical engineering applications. Therefore, it is difficult to design practical filters with favorable feature recognition for generalized multiple-impact signals [13,14].

For multiple mechanical impact loads with repetitive impulses, deep learning is another enticing technical route with the potential to address such problems, achieving relatively high identification accuracy without difficult filter designs [15]. Deep learning achieves much more abstracted and advanced signal features through the construction of multiple-layer simple nonlinear modules and improves the accuracy of signal classification [16]. Researchers have reported the application of deep learning in identifying spacecraft overload and other complex signals, with accuracies exceeding those of traditional methods. For example, Ibrahim S K designed an LSTM-based network to identify multiple impact loads even when the locations of the spacecraft’s load impacts are unknown [17]. Zhou et al. proposed improved LSTM and GRU networks and used them to identify multiple signals based on actual telemetry data from satellites to achieve spacecraft fault diagnosis and classification [18].

Although a variety of deep learning-based signal feature recognition methods have been developed, there are still some technical shortcomings in relation to their application in dynamic multi-impact scenarios. First, traditional deep learning algorithms are complex and have longer time delays in signal processing. This is an Achilles’ heel for ammunition penetration and other multiple impact applications, which involve real-time decisions since a 5-millisecond time delay can result in a detonation error of more than 5 m for ammunition, leading to a significant reduction in damage effectiveness [19]. In addition, in spacecraft and many other onboard and military systems, which are constrained by the size of the space, it is only possible to have microcontrollers with weak hardware resources rather than advanced processors with a larger size and higher performance, making it impossible for overly complex deep learning algorithms to run [20]. Therefore, it is urgent and of practical value to design lightweight deep learning-based multiple-impact signal processing algorithms.

To address this urgent need, we propose an algorithm that combines a wavelet transform preprocess with lightweight, elaborately designed fast-activated minimal gated unit (FMGU) neural networks, which significantly reduces computational complexity while maintaining high identification accuracy. Comprehensive performance validation was conducted under different multi-impact datasets with various complexities, including those with ultra-high impact speeds and ultra-strong noise.

Our scientific contributions are summarized as follows. Firstly, we have simplified the complex physical process of multiple impacts and proposed its dynamics model. Based on this model, we analyzed the features and coupling relationships between signals and noise interference, which provides a foundation for designing a deep learning network with high recognition accuracy. Secondly, we conducted a wavelet time-frequency domain transform of multiple impact signals. By calculating layer coefficients and interlayer coefficients, we evaluated the suppression of signal adhesion after wavelet transform preprocessing, and this suppression made it possible to realize key feature recognition via lightweight networks. Finally, we modified the gate unit structure and activation function of the traditional GRU network and proposed FMGU, which reduces 50% of the computation and 75% of the latency while causing no degradation in the recognition accuracy.

## 2. Transfer Model for Multiple Impacts in Multibody Dynamical Systems

**Figure 1:**
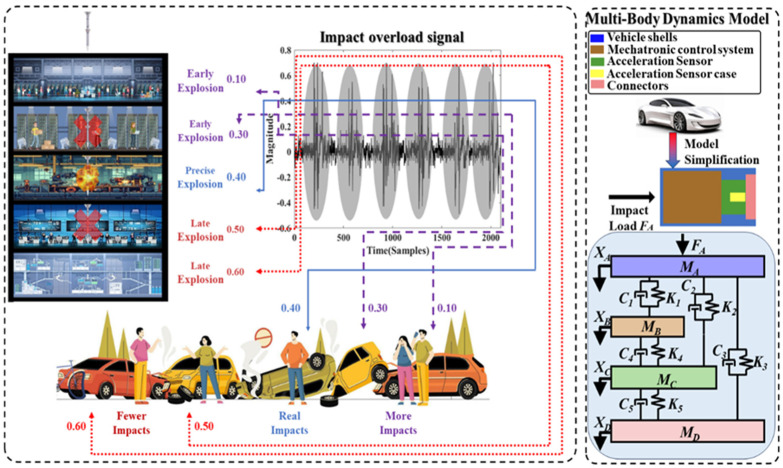
Spring-damping equivalent model for multiple impact processes.

In the actual impact process, the multiple impact signals measured by the acceleration sensor are superimposed with significant nonlinear noise and interference due to high-speed friction between the car and the target, structural damage of the vehicle, and complex stress transfer between internal vehicle components [21,22,23]. Similar to studies in many other fields, we established a spring-damping equivalent model for multiple impact processes, as shown in Figure 1. The model equations are shown in (1)–(4).

(1) $$M_{A}\ddot{x_{A}}=p_{1}\left(x_{A},x_{B}\right)+p_{2}\left(x_{A},x_{C}\right)-F_{A}$$

(2) $$M_{B}\ddot{x_{B}}=K_{4}\left(x_{B}-x_{C}\right)+C_{4}\left(\dot{x_{B}}-\dot{x_{C}}\right)-p_{1}\left(x_{A},x_{B}\right)$$

(3) $$M_{C}\ddot{x_{C}}=K_{5}\left(x_{C}-x_{D}\right)+C_{5}\left(\dot{x_{C}}-\dot{x_{D}}\right)-K_{4}\left(x_{B}-x_{C}\right)-C_{4}\left(\dot{x_{B}}-\dot{x_{C}}\right)-p_{2}\left(x_{A},x_{C}\right)$$

(4) $$M_{D}\ddot{x_{D}}=K_{3}\left(x_{C}-x_{D}\right)+C_{3}\left(\dot{x_{C}}-\dot{x_{D}}\right)-K_{5}\left(x_{C}-x_{D}\right)-C_{5}\left(\dot{x_{C}}-\dot{x_{D}}\right)$$

where $M_{A},\;M_{B},\;M_{C}$, and $M_{D}$ are the masses of the vehicle shell, mechatronic control system, acceleration sensor, and connectors, respectively; $x_{A}$, $x_{B}$, $x_{C}$, and $x_{D}$ are the corresponding displacements of the different components; $K_{1}$, $K_{2},\;K_{3}$, $K_{4}$, and $K_{5}$ are stiffness coefficients. $C_{1},\;C_{2}$, $C_{3}$, $C_{4}$, and $C_{5}$ are damping coefficients; $F_{A}$ is the dynamic impact force.

(5) $$p_{1}\left(x_{A},x_{B}\right)=\left\{\begin{array}{r} K_{1}\left(x_{A}-x_{B}-d_{1}\right),\;x_{A}-x_{B}>d_{1}\\ 0,\;\left|x_{A}-x_{B}\right|⩽d_{1}\\ K_{1}\left(x_{A}-x_{B}-d_{1}\right)+C_{1}\left(\dot{x_{A}}-\dot{x_{B}}\right),\;x_{A}-x_{B}<-d_{1} \end{array}\right.$$

(6) $$p_{1}\left(x_{A},x_{C}\right)=\left\{\begin{array}{r} K_{2}\left(x_{A}-x_{C}-d_{1}\right)+C_{2}\left(\dot{x_{A}}-\dot{x_{C}}\right),\;x_{A}-x_{C}>d_{1}\\ 0,\;\left|x_{A}-x_{C}\right|⩽d_{1}\\ K_{1}\left(x_{A}-x_{B}-d_{1}\right)+C_{2}\left(\dot{x_{A}}-\dot{x_{C}}\right),\;x_{A}-x_{C}<-d_{1} \end{array}\right.$$

In the spring-damping equivalent model, $p_{1}\left(x_{A},x_{B}\right)$, $p_{2}\left(x_{A},x_{C}\right)$, and $p_{3}\left(x_{A},x_{E}\right)$ represent the non-ideal forces between different components. These are caused by $d_{1},\;d_{2}$, and $d_{3}$, which are the gap distances between the vehicle shell and the other three components. Specific relationships are shown in Formulas (5) and (6).

This multi-body dynamics modeling method is applicable to both types of scenarios. However, the specific numbers of springs and dampers and their connection relationships need to be determined based on the component count and connections in the actual system case by case. The precise values of equivalent stiffness and damping must be calibrated through finite element analysis [24]. For automotive multiple impacts and projectile penetration scenarios, the system comprises numerous components with a complex mechanical transmission process, and there is significant nonlinear noise present, which can affect the accurate identification of impact counts.

From the above equivalent model, it is evident that the mechanical transfer process of multiple impacts, especially under high-speed conditions, is very complex and can be affected by damping, stiffness, gap distances, and many other uncertain parameters. Thus, the signal measured by an accelerometer sensor contains strong high-frequency oscillatory interference, and it is difficult to identify the original impact features. A multiple-impact signal is shown in Figure 1, and an accurate judgment of the number of impacts cannot be made using thresholds only. If threshold lines of 0.10 or 0.30 are used as criteria, more impacts than are actually present will be detected; if threshold lines of 0.5 or 0.6 are used as criteria, fewer impacts than are actually present will be detected. If 0.4 is chosen as a threshold, the number of impacts can be correctly identified, and it lacks generalizability for judging other signals.

## 3. Combined Algorithm of Wavelet Transform Preprocess and FMGU Network

**Figure 2:**
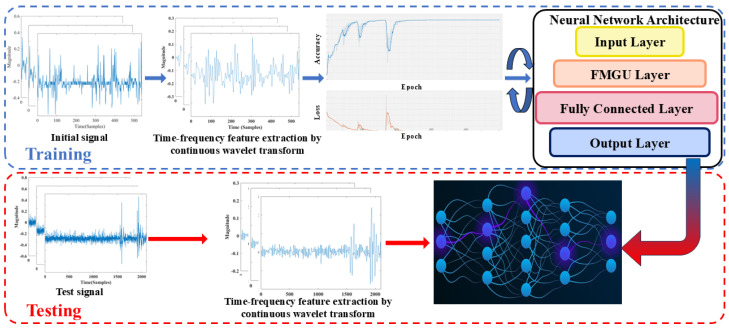
Technological route for FMGU network training and testing.

To address the issue of identifying multiple impact signals accurately and quickly, we propose an algorithm that combines wavelet transforms with lightweight neural networks. After wavelet preprocessing, the signal adhesion phenomenon can be significantly reduced, enabling key feature recognition via simpler neural networks. In terms of network design, we propose FMGU, which simplifies the structure of the update and reset gate units in traditional GRU networks [25,26]. Additionally, the relatively complex sigmoid and tanh functions are replaced with linear function approximations, further reducing computational complexity. The specific technical route of the study is shown in Figure 2. Firstly, we obtain multi-impact signals with different impact numbers and speeds by experiment, including ultra-high-speed impact cases. Secondly, we perform wavelet transform as a preprocess to reduce the signal adhesion phenomenon of multi-impact signals. Thirdly, the FMGU network is trained while continuously adjusting parameters to achieve the highest recognition accuracy. Finally, we compare FMGU’s identification accuracy and some other metrics with other widely used networks to verify FMGU’s superior performance.

Wavelets are commonly used functions for analyzing signals in the time-frequency domain [27]. They involve a special type of function with finite duration and limited oscillation numbers, which begin with an amplitude of zero and finally return to zero. These functions are concentrated around a specific point, with an integral value of zero. The wavelet transform formula is shown in Formula (7).

(7) $$W_{f}\left(a,b\right)=\langle f,\varphi_{a,b}\rangle ={\left|a\right|}^{-\frac{1}{2}}\int f\left(t\right)\bar{\varphi \left(\frac{t-b}{a}\right)}dt$$

where $\varphi_{a,b}$ represents a continuous wavelet with scaling parameter a and translation parameter b, where a, b ∈ R (the set of real numbers) and a ≠ 0. The parent wavelet φ changes its shape by adjusting the scaling factor a, and its position in time is shifted by adjusting the translation factor b. $\langle f,\varphi_{a,b}\rangle$ is the inner product of two functions. $\bar{\varphi }$ is the complex conjugate of φ. By adjusting these scaling and translation parameters, local analysis in time and frequency can be performed to help analyze the instantaneous frequency changes and local features of signals.

By using a series of wavelet functions to decompose and reconstruct signals, it is possible to analyze signal features at various scales and frequencies. This approach can also capture local abrupt changes in time and frequency, making it particularly effective for analyzing and processing mutation signals. Wavelet transform effectively reduces the signal adhesion of multiple impacts, which makes it possible to realize key feature recognition via lightweight deep learning-based networks. Choosing a higher number of decomposition levels can decompose the signal to a more detailed frequency level and separate the signal and noise more effectively; the number of decomposition levels in this paper is 4. Furthermore, this paper uses the db4 wavelet as the mother wavelet for wavelet transformation. It has good high-frequency characteristics at higher orders, making it suitable for extracting high-frequency details while maintaining high computational efficiency. The scale is the same for different numbers of impact signals, which ensures fair analysis and comparison of their time-frequency domains. Specifically, the scale is set to half the number of sample points, which is the usual practice in wavelet transform.

**Figure 3:**
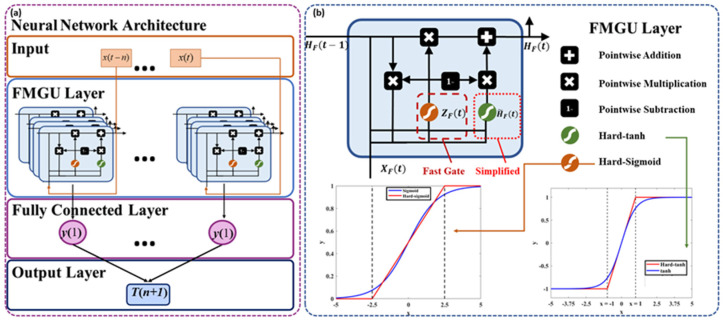
FMGU neural network: (**a**) FMGU network architecture; (**b**) FMGU layer architecture.

The overall network structure is shown in Figure 3a. The network takes the signal features extracted after wavelet transformation as input, passes them through the FMGU layer and into a fully connected layer, and finally outputs the classification results from the output layer. FMGU was developed by improving GRU networks, which are currently some of the most widely used lightweight networks so that we can ensure that the algorithm’s complexity is low enough to run in real-time on microcontrollers with limited hardware resources.

GRU contains an update gate and a reset gate, where the update gate is used to control the cell state update, and the reset gate decides how to combine the new input information with the previous memory [28,29,30]. It exhibits good performance in identifying time-series data signals. However, it has a large number of network parameters, a relatively complex structure, and a slow computational speed, requiring powerful hardware for computational support [31,32,33,34].

To address these issues, FMGU further reduces the network unit parameters, time delay, and training time compared to traditional time-series models, as confirmed by our experiments on cases in Section 4. Compared to GRU, a fast gate combines the functions of the update and reset gates in the GRU, allowing the model to reduce the parameters while still effectively capturing temporal dependencies. The main input unit structure is shown in Figure 3b. In addition, to further reduce the network time delay and enhance hardware compatibility, modifying the activation functions is employed to minimize computational costs. This approach can be used to further realize the demand for lightweight networks to process information quickly. The update method of the fast gate is shown in Formula (8).

(8) $$Z_{F}\left(t\right)=\sigma_{h}\left(W_{F}\cdot \left[H_{F}\left(t-1\right),X_{F}\left(t\right)\right]+b_{F}\right)$$

where $Z_{F}\left(t\right)$ is the output of the fast gate, and $W_{F}$ and $b_{F}$ are the weight value and bias value of the fast gate, respectively. $X_{F}\left(t\right)$ is the input at time t, and $H_{F}(t-1)$ is the hidden state value at time t−1. This gating mechanism determines the weight of information from the previous moment step and the current moment step in updating the current hidden state. It resembles a combination of the update and reset gates in GRU but within a more simplified framework. The hard-tanh function is used in the fast gate, and $\sigma_{h}$ is the hard-sigmoid function; these are calculated as follows:

(9) $$hard-tanh\left(x\right)=\left\{\begin{array}{l} -1,\;x<-1\\ \;x,-1\leq x\leq 1\\ \;1,\;x>1 \end{array}\right.$$

(10) $$\sigma_{h}\left(x\right)=\left\{\begin{array}{c} \;0,\;x<-2.5\\ 0.2x+0.5,-2.5\leq x\leq 2.5\\ 1,\;x>0 \end{array}\right.$$

In theory, the closer the segmentation function is to the sigmoid function, the more accurate the fitting effect is, but this also increases computational complexity and reduces efficiency. To balance the two performances, we designed it as a function in three segments. Based on the update method of the fast gate, the state update proceeds as follows:

(11) $$\tilde{H_{F}}\left(t\right)=hard-tanh\left(W_{h}\left[R_{F}\left(t\right)\cdot H_{F}\left(t-1\right),X_{F}\left(t\right)\right]+b_{F}\right)$$

(12) $$H_{F}\left(t\right)=Z_{F}\left(t\right)\cdot H_{F}\left(t-1\right)+\left(1-Z_{F}\left(t\right)\right)\cdot \tilde{H_{F}}\left(t\right)$$

where $\tilde{H_{F}}\left(t\right)$ is the output of the candidate’s hidden state; $H_{F}\left(t\right)$ is the output of the system’s hidden state. Candidate hidden states combine new input information with past information to provide possible new values for updating the system’s hidden state. The hidden state integrates the state information from a previous moment and the information from the candidate hidden state, which can further be adjusted by the parameters in the update gate to ensure the balance between the old and new information.

## 4. Results and Discussion

In this study, we obtained original multiple-impact signals through standardized multi-impact testing equipment. Since we were only concerned with the identification of the number of impacts, we normalized the amplitude values, and the specific experimental methods are shown in Appendix A. Specific multiple impact experimental methods are in the Supplementary Materials. Based on the experimental data, we calculated metrics such as the classification accuracy, computational complexity, and time delay of the FMGU network. Comparison with networks like LSTM and GRU demonstrated that the FMGU network achieves high accuracy in dealing with problems while maintaining lightweight characteristics. The hardware specifications used in this experiment were as follows: CPU: 13th Gen Intel(R) Core (TM) i9-13980HX; GPU 0: Intel(R) UHD Graphics; GPU 1: NVIDIA GeForce RTX 4060 Laptop GPU.

### 4.1. Contributions of Wavelet Preprocessing and FMGU Network

**Figure 4:**
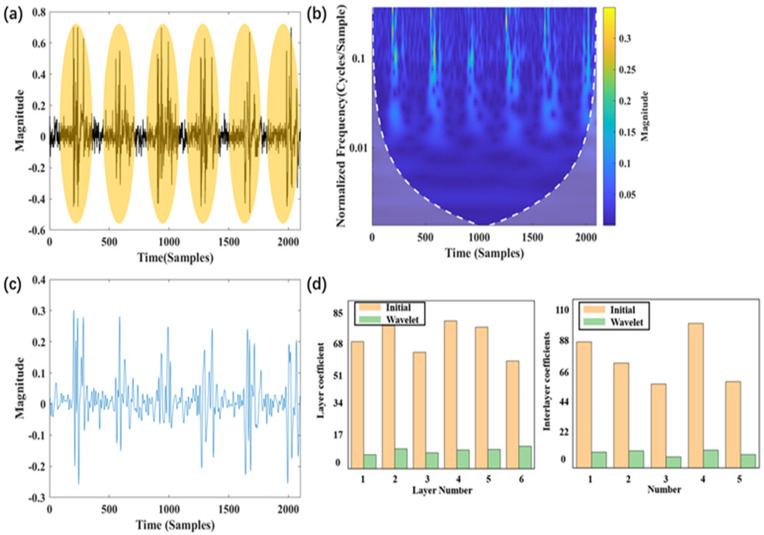
Features of low-speed signal: (**a**) original data; (**b**) time-frequency domain transform; (**c**) reconstructed signal; (**d**) layer and interlayer coefficients.

**Figure 5:**
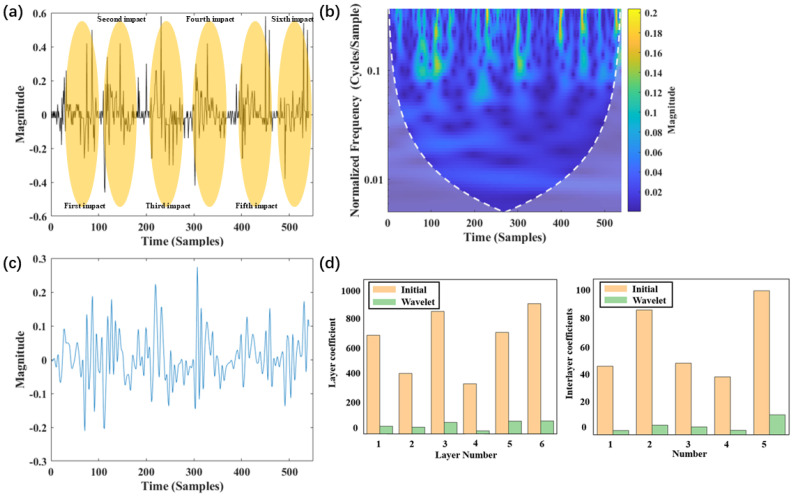
Features of high-speed signal: (**a**) original data; (**b**) time-frequency domain transform; (**c**) reconstructed signal; (**d**) layer and interlayer coefficients.

Figure 4a and Figure 5a show the original signals of six impacts at low and high speeds (250 m/s and 1000 m/s), respectively. From the signal graph, it can be observed that when the impact speed is low, the signal adhesion phenomenon is not very severe. This allows for relatively easy identification of impact numbers. However, as the impact speed increases, the signal adhesion phenomenon becomes very pronounced, making it difficult to distinguish between multiple impact conditions. Figure 4b and Figure 5b show the time-frequency domain features extracted using wavelet transformation. Under low-speed conditions, the time-frequency domain graph exhibits clear, discrete arrays of patterns. Conversely, under high-speed conditions, the time-frequency domain graph does not exhibit clear patterns. Figure 4c and Figure 5c show the reconstructed signal after wavelet transformation. It is clear that the interference is suppressed. Figure 4d and Figure 5d demonstrate the layer coefficients and interlayer coefficients of multiple impact signals both before and after wavelet preprocessing, and they are important metrics for characterizing the significance of signal adhesion. Both metrics show an order of magnitude decrease, verifying the contribution of wavelet preprocessing to the identification of impact features. Notably, for Figure 5d, although wavelet transformation has significantly reduced interference, the layer coefficients and interlayer coefficients remain relatively large. This is the reason why direct identification of the impact number cannot be achieved solely via wavelet transformation.

**Figure 6:**
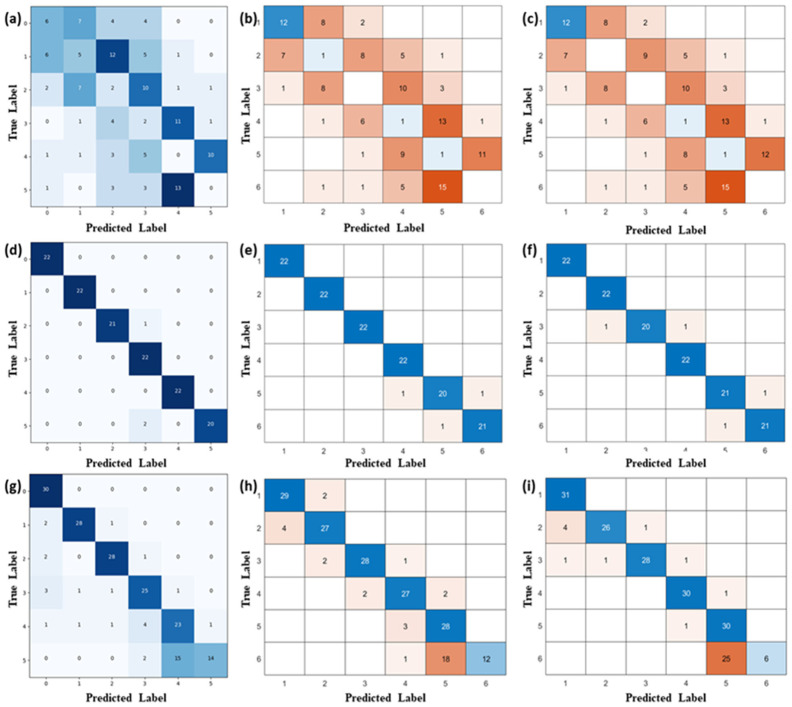
Identification accuracy results: (**a**–**c**) identification results of the original signal for FMGU, LSTM, and GRU networks; (**d**–**f**) identification results of wavelet-transformed low-speed signals for FMGU, LSTM, and GRU networks; (**g**–**i**) identification results of wavelet-transformed high-speed signals for FMGU, LSTM, and GRU networks.

Wavelet preprocessing not only contributes significantly but is also indispensable. LSTM and GRU are highly adaptive to pattern identification at different time scales and have excellent universality [35,36,37,38]. Figure 6a–c demonstrates the recognition performance of multiple impact counts for the FMGU, LSTM, and GRU networks without wavelet preprocessing, respectively. These three networks cannot accurately distinguish the number of impacts with a test accuracy of less than 30%, especially when the impact number further increases. Figure 6d–f depicts the test results of three networks with the data after wavelet preprocessing as input. At low impact speeds, the signal adhesion phenomenon is not strong, and the networks all have very high signal identification accuracies. LSTM and FMGU have identification accuracies of 98%, while GRU’s accuracy is 97%. It is evident that the classification accuracy has substantially improved.

Figure 6g–i further shows the identification accuracies of the three networks for high-speed impact signals, respectively. The increase in speed results in an increase in nonlinear interference, and the identification performance of the number of impacts of several methods decreases significantly. The identification accuracy is 80% for FMGU, 82% for LSTM, and 81% for GRU. As the number of impacts increases, the interference of the signal is aggravated, which makes feature identification of impacts much more difficult. The identification accuracy at 5 and 6 times the number of impacts is obviously lower than that at 1 and 2 times the number.

Combining the recognition accuracy results of low-speed and high-speed signals, the proposed algorithm, which combines wavelet transforms with lightweight FMGU neural networks, is suitable for the real-time feature recognition of multiple impact signals. The lightweight FMGU network is comparable to LSTM and GRU networks in terms of recognition accuracy even though the number of gates is reduced, and linear function approximations are used to replace the activation functions, which are relatively complex to compute.

### 4.2. Robustness of FMGU Network Verification

For deep learning algorithms, the robustness of recognition accuracy is important, determining whether the algorithm is effective when subjected to new datasets it has not learnt before. Robustness is especially critical for applications in vehicle crash testing, penetrating munition fuses, and so on, where the slightest misrecognition can lead to serious safety issues [39,40,41].

**Figure 7:**
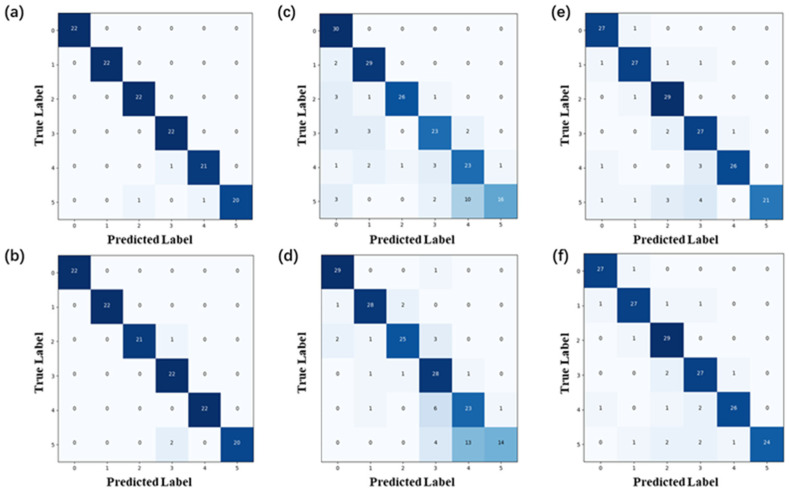
Robustness of FMGU network verification: (**a**,**b**) multiple identification results of low-speed signals; (**c**,**d**) multiple identification results of high-speed signals; (**e**,**f**) multiple identification results of high- and low-speed mixed signals.

The datasets used in Figure 7a–f comprise low-speed data, high-speed data, and mixed data, respectively. When dealing with low-speed signals, identification accuracy is 97% in these two cases, which is relatively stable. It is more difficult to identify high-speed impact signals, with recognition accuracies of 77.3% and 77.8%. For the experiments with mixed data, recognition accuracies are 88.2% and 89.8%. In conclusion, the system can still maintain a relatively stable accuracy when the data pattern is changed appropriately. The network proposed in this paper is robust and of great importance for engineering applications.

### 4.3. Comparison of Real-Time Performance and Hardware Overhead Analysis

The real-time performance and hardware overhead of multiple impact signal characterization methods are also important for applications such as vehicle impact testing and penetrating munition fuses. The difference in real-time performance is basically due to different floating point operations (FLOPs) that result from differences in the internal computing architectures of the FMGU, GRU, and LSTM networks [42]. For example, GRUs are updated as follows:

(13) $$z\left(t\right)=\sigma \left(W_{Z}\cdot \left[h\left(t-1\right),x\left(t\right)\right]+b_{z}\right)$$

(14) $$r\left(t\right)=\sigma \left(W_{r}\cdot \left[h\left(t-1\right),x\left(t\right)\right]+b_{r}\right)$$

(15) $$\tilde{h\left(t\right)}=tanh\left(W_{h}\cdot \left[r\left(t\right)⊙h\left(t-1\right),x\left(t\right)\right]+b_{h}\right)$$

(16) $$h\left(t\right)=\left(1-z\left(t\right)\right)⊙h\left(t-1\right)+z\left(t\right)⊙\tilde{h\left(t\right)}$$

**Figure 8:**
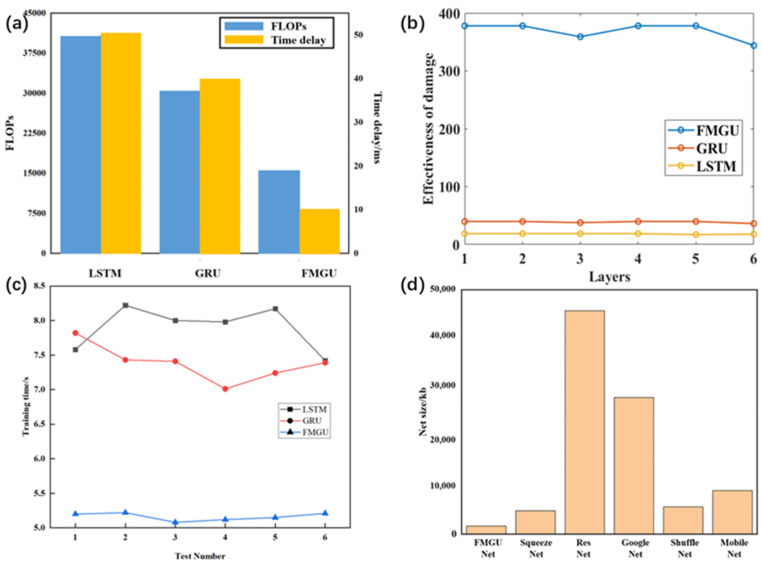
Comparison of real-time performance and hardware overhead analysis: (**a**) comparison of time delay and computational complexity; (**b**) comparison of damage effectiveness; (**c**) comparison of training time; (**d**) comparison of FMGU’s execution file size with several typical lightweight networks.

Since FMGU has only one gate that combines the functions of updating and resetting gates in the GRU, it does not need to perform the operations of Formula (13), resulting in lower FLOPs compared to GRU. Figure 8a shows the comparison of network FLOPs and time-delay data for the three networks. In the same situation, the number of FLOPs for FMGU is reduced by 63% compared to LSTM and 50% compared to GRU, which shows it is much lower in computational complexity than the above networks. The time delay of FMGU is only 10 ms, while LSTM takes 50 ms and GRU takes 40 ms. This reduces time delay by 80% compared to LSTM and 75% compared to GRU. The above results illustrate that FMGU substantially improves real-time performance in various application scenarios and can achieve a fast response and efficient execution.

Notably, two of the most critical factors affecting the degree of damage in penetrating ammunition are the accuracy of feature recognition and the time delay. The accuracy of the recognition directly determines whether the explosion can be carried out after a pre-set number of layers, and the time delay determines the distance between the actual explosive point and the pre-set explosive point. Damage effectiveness can be characterized quantitatively using the following formula [43,44]:

(17) $$V=k_{d}\left(V_{s}e^{-k_{a}\left(V_{d}\cdot t_{s}\right)}\right)\cdot \varepsilon$$

where $k_{d}=1$ is the damage efficiency coefficient, $V_{d}=250$ m/s is the velocity of detonation, $k_{a}=0.7$ is the attenuation coefficient, $V_{s}=1000$ m/s is the velocity of ammunition, ε is the accuracy of identification, and $t_{s}$ is the time delay.

We used the identification accuracy and network time delay in high-speed experiments to calculate the damage effectiveness of the three networks for different numbers of layers, as shown in Figure 8b. The damage effectiveness of FMGU is about 9.7 times that of GRU and 20.1 times that of LSTM in different layers. Such huge differences in damage effectiveness are due to the fact that, although the feature recognition accuracy of FMGU for multiple impact signals is roughly comparable to those of GRU and LSTM, the time delay is much shorter than that for both of them. The distance between the actual explosive point and the pre-set explosive point is only 10 m for FMGU with a delay of 10 ms, whereas GRU and LSTM reach 40 m and 50 m, respectively.

Figure 8c compares the training times required for the three networks. The training times for LSTM and GRU are both longer than that required for FMGU. FMGU requires 69% of the time needed by GRU and 65% of LSTM, illustrating its lower hardware overhead.

Moreover, in addition to the above parameters, the size of the execution file is also an important metric for evaluating whether the model can run on weak hardware resources with limited computational capability. Figure 8d shows a comparison of the size of several commonly used lightweight network models. Among them, Squeeze Net and Shuffle Net are widely known for their lightweight nature. However, their model files are still relatively large compared to FMGU. Excitingly, FMGU’s network file size is only 35% of Squeeze Net’s, making it perfect for deployment on weak hardware resources.

## 5. Conclusions

We have proposed an algorithm that combines wavelet transforms with lightweight, elaborately designed FMGU neural networks, which significantly reduces computational complexity while maintaining high identification accuracy. First, we proposed the FMGU network, which simplifies the structure of the update and reset gate units in traditional GRU networks and replaces the complex sigmoid and tanh functions with linear function approximations, achieving high accuracy in signal classification while remaining lightweight. The combination of FMGU with wavelet preprocessing is suitable for the feature recognition of multiple impact signals. Secondly, we obtained original multiple impact signal datasets through standardized mechanical impact equipment, including those with ultra-high impact speeds. After testing, the feature recognition accuracy of the proposed FMGU-based method was comparable to that of LSTM and GRU, while the time delay was 75% shorter than that of GRU and 80% shorter than that of LSTM. Third, the training time of the proposed method is short enough, and the execution file is small enough to be favorable for running on real hardware for penetration ammunition, with damage effectiveness of 9.7 times that of GRU and 20.1 times that of LSTM. Overall, the proposed method is a new solution for multiple-impact signal recognition, balancing high recognition accuracy and low computational complexity, which demonstrates its practical value in penetrating ammunition and other application platforms with weak hardware.

## Data Availability

Data of this work is unavailable due to privacy.

## Supplementary Materials

In this study, we obtained original signals through standardized multi-impact testing equipment. The supporting information of multiple impact can be downloaded at: https://www.mdpi.com/article/10.3390/s24165245/s1, Figure S1: Multiple impact equipment. In this study, we obtained original signals through standardized multi-impact testing equipment.

## Appendix A

### Appendix A.1. Multiple Impact Signal Generation

The multiple impact data for the experiment described in this paper was generated by standardized multiple impact test equipment, as shown in references [45,46,47]. The signals were divided into two categories, low speed, and high speed, and the number of impacts was 1–6 times. The workflow was as follows. We calculated the required rotation speed according to the impact interval requirements and set the vibration loading parameters based on experimental needs. Once the rotation accelerated to the set speed, vibration loading began. Multiple high-speed rotating impact components sequentially made contact with the test fixture to deliver repeated impact loads to the test specimen. After completing the specified number of impacts, the hydraulic system controlled the overall retraction of the impacted components, retracting the motion path of the impact head and stopping vibration loading. Equivalent test conditions consisted of simulating low- (250 m/s) and high-speed (1000 m/s) penetration processes, while the data acquisition system was responsible for collecting and storing generated datasets. There were 330 datasets for the low-speed condition and 420 for the high-speed condition. For the high-speed set, the number of samples in the test set is 185. For the low-speed set, the number of samples in the test set is 132.

### Appendix A.2. Evaluation of Signal Adhesion

We evaluated the interference superposition level in the signal target features using layer and interlayer coefficients as evaluation metrics [48]. These metrics not only measure the degree of interference adhesion in acceleration signals but also provide a quantitative evaluation of the processing effect of filtering algorithms. For the same multiple impact signal, the lower the layer and interlayer coefficients are, the better the algorithm is. Inputs for calculation included the amplitude of the acceleration signal, signal sampling frequency, number of impacts, and time interval between impacts in each layer. The method for calculating the layer coefficients $a_{i}$ is shown in Formula (A1), and the interlayer coefficients ${ac}_{i,i+1}$ were calculated using Formula (A2).

(A1) $$a_{i}=\frac{\sum_{k=1}^{N_{i}}\left|a_{i}\left(k+1\right)-a_{i}\left(k\right)\right|}{N_{i}T_{i}}$$

(A2) $${ac}_{i,i+1}=\frac{\sum_{k=N_{i}/2}^{N_{i}-1}\left|a_{i}\left(k+1\right)-a_{i}\left(k\right)\right|+\sum_{k=1}^{N_{i+1}/2}\left|a_{i+1}\left(k+1\right)-a_{i+1}\left(k\right)\right|+\left|a_{i+1}\left(1\right)-a_{i}\left(N_{i}\right)\right|}{\left(N_{i}T_{i}+N_{i+1}T_{i+1}\right)/2}$$

where $a\left(t\right)$ is the acceleration signal $\left(g\right)$, $a\left(n\right)$ is the result of sampling $\left(g\right)$, L is the number of impacts, $T_{S}$ is the sampling period of the signal (ms), $N_{1},N_{2}$, ⋯, $N_{L}$ is the number of sampling points for each impact, and $T_{1},T_{2}$, ⋯, $T_{L}$ is the duration of each impact acceleration signal. The number of points and signal duration satisfy $T_{i}=N_{i}\cdot T_{S}$, where i = 1, 2, ⋯, L. The whole signal duration is $T=N\cdot T_{S}$, where N is the total number of data points. Finally, signal data are segmented, $a_{1}\left(n\right)=a\left(n\right),n\in \left[1,N_{1}\right]$; $a_{2}\left(n-N_{1}\right)=a\left(n\right)$, $n\in \left[N_{1}+1,N_{1}+N_{2}\right]$.
