# Supplementary material for: Fast-Activated Minimal Gated Unit: Lightweight Processing and Feature Recognition for Multiple Mechanical Impact Signals
Source: Sensors (Basel). 2024 Aug 14;24(16):5245. doi: 10.3390/s24165245 (PMC11359475; doi:10.3390/s24165245)
Supplement: Supplementary file 1 [file sensors-24-05245-s001.zip › sensors-3132607-supplementary.pdf]

Manuscript ID: sensors-3132607

Title: Fast-activated Minimal Gated Unit: Lightweight Processing and Feature Recognition for Multiple Mechanical Impact Signals

Author name: Wenrui Wang, Dong Han, Xinyi Duan, Yaxin Yong, Zhengqing Wu, Xiang Ma, He Zhang and Xiaofeng Wang

Below is a specific explanation of multiple impact. Taking a standard impact-vibration coupling test as an example, the workflow of the mechanical testing system is described as follows[1]:

1) Before the test begins, first install the test specimen and the impact signal acquisition module, and according to the simulation needs of the self-excited vibration particles in the cavity of the granular body into the particulate body. Start the air compressor, hydraulic station, and other equipment to provide the necessary air pressure and hydraulic sources for system operation.

2) Power on the system and activate the control system. Perform a self-check of the functional modules, which includes verifying the initial position of the feed mechanism using position sensors, and checking the oil pressure of the hydraulic feed system and the air pressure of the pneumatic clutch using pressure sensors to ensure they meet operational requirements. Throughout the test, the control system continuously monitors the system conditions in real-time and responds promptly to fault signals.

3) Based on the required test conditions for simulation, calculate the corresponding impact intervals, active excitation loading frequency, and total number of impacts, and set these parameters via the touch screen.

4) Start the motor, and the turntable begins to rotate and accelerate. Due to the large mass of the turntable system, a gradual acceleration curve is set via the frequency converter, and the turntable speed is stabilized using speed feedback control.

5) Once the turntable reaches the set speed, the active excitation loading device begins operation, and the hydraulic feed system is activated to move the test specimen to the impact position and start the impact test. Meanwhile, the feed mechanism's start pulse is used as a trigger signal to synchronize the signal acquisition system for collecting acceleration data.

6) After completing the specified number of impacts, the control system sends a trigger signal to stop the active excitation loading and enable the hydraulic feed system to drive the test specimen back to the initial position, ceasing the impacts. Simultaneously, the trigger signal synchronizes the cessation of impact acceleration signal acquisition, and the turntable slows down and brakes.

7) Read the impact test data via the touch screen or the upper-level computer, and retrieve the test samples for further structural analysis and other evaluations.

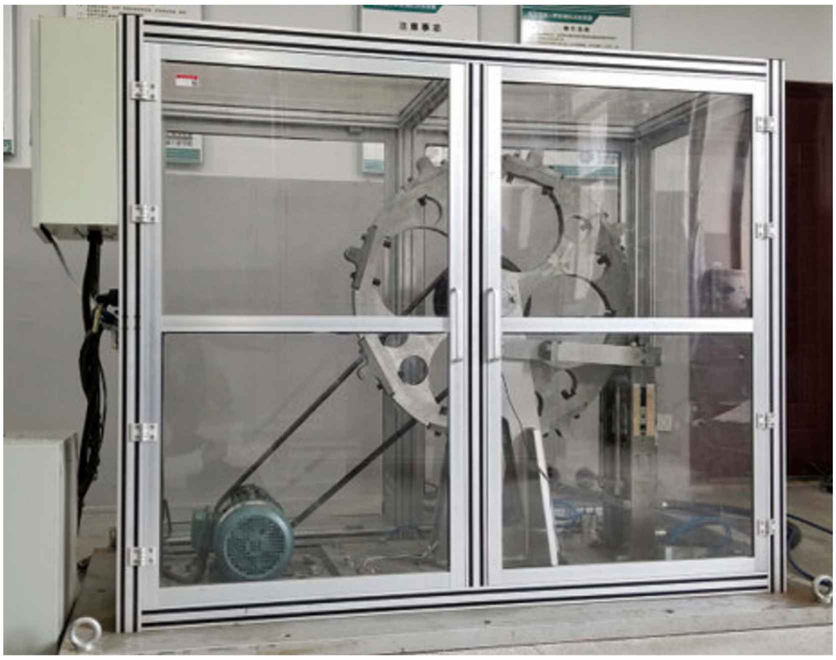

Figure S1. Multiple impact equipment.[1][2]

The following provides an introduction to the principles of the signal acquisition device.

To achieve the acquisition of impact and vibration test data and the evaluation of test results, the mechanical testing system is equipped with an online signal acquisition device. This device mainly includes a power supply module, signal conditioning circuit, A/D conversion circuit, FPGA main control circuit, storage module, and communication module. [1]

The front end of the signal acquisition device consists of an accelerometer, which converts impact acceleration into an electrical signal through its sensing element. This signal is then differentially amplified, zeroed, and filtered by the signal conditioning circuit, and subsequently sent to the FPGA main control chip through the A/D conversion module for processing and storage in the storage module. [3]

To meet the data acquisition and storage requirements for high-dynamic impact tests in this system, the storage module uses SRAM memory with high-speed storage and retrieval capabilities for temporarily storing test data. After the impact test is completed, the main FPGA control unit responds to the touch screen's data reading command and uploads the test data to the touch screen for display and storage through the communication module.

The software functions of the signal acquisition device can be divided into two main parts: signal acquisition and signal reading. After powering on and initializing, the FPGA main control circuit will await commands from the touch screen for either signal acquisition or signal reading and will enter the corresponding operating mode. When configured for signal acquisition mode, the FPGA control chip will perform high-speed cyclic sampling of the A/D module and check whether the output signal from the accelerometer is normal. If the output signal is abnormal, an alarm message will be generated. If the signal is normal, the system will perform threshold checking. When acceleration data meeting the threshold conditions is continuously received, the

program automatically determines that the impact test has begun and will collect and store test data before and after the trigger moment. The data collection will continue until a feed/retraction trigger signal is received, indicating the end of the impact test, at which point signal acquisition and storage will stop. When the signal acquisition device is set to signal reading mode, the FPGA control circuit will respond to the corresponding commands to complete the reading and transmission of data from the storage chip.

References

[1]. Li F. Research on the theory and method of multiple short interval impact-vibration coupling mechanical test[D]. Jiangsu: Nanjing University of Science and Technology,2021.

[2]. Li F, Ma S. Analysis and experimental study of acceleration model for short interval and multiple impact equipment[J]. Shock and Vibration, 2019, 2019(1): 5139137.

[3]. Li F, Ma S. Design of multiple rotating impact experiment system of high speed and heavy overload[C]//2017 3rd IEEE International Conference on Control Science and Systems Engineering (ICCSSE). IEEE, 2017: 183-187.
